# A Scoping Review on Minimum Foot Clearance Measurement: Sensing Modalities

**DOI:** 10.3390/ijerph182010848

**Published:** 2021-10-15

**Authors:** Ghazaleh Delfi, Abdulrahman Al Bochi, Tilak Dutta

**Affiliations:** 1 KITE—Toronto Rehabilitation Institute, University Health Network, Toronto, ON M5G 2A2, Canada; ghazaleh.delfi@mail.utoronto.ca (G.D.); aalbochi@ryerson.ca (A.A.B.); 2 Institute of Biomedical Engineering, University of Toronto, Toronto, ON M5S 3G9, Canada

**Keywords:** minimum foot clearance, minimum toe clearance, older adults, falls, tripping, prevention

## Abstract

*Background:* Falls are a major public health issue and tripping is the most common self-reported cause of outdoor falls. Minimum foot clearance (MFC) is a key parameter for identifying the probability of tripping. Optical motion capture systems are commonly used to measure MFC values; however, there is a need to identify alternative modalities that are better suited to collecting data in real-world settings. *Objective:* This is the first of a two-part scoping review. The objective of this paper is to identify and evaluate alternative measurement modalities to optical motion capture systems for measuring level-ground MFC. A companion paper identifies conditions that impact MFC and the range of MFC values individuals that these conditions exhibit. *Methods:* We searched four electronic databases, where peer-reviewed journals and conference papers reporting level-ground MFC characteristics were identified. The papers were screened by two independent reviewers for inclusion. The reporting was done in keeping with the PRISMA-ScR reporting guidelines. *Results:* From an initial search of 1571 papers, 17 papers were included in this paper. The identified technologies were inertial measurement units (IMUs) (*n* = 10), ultrasonic sensors (*n* = 2), infrared sensors (IR) (*n* = 2), optical proximity sensors (OPS) (*n* = 1), laser ranging sensors (*n* = 1), and ultra-wideband sensors (*n* = 1). From the papers, we extracted the sensor type, the analysis methods, the properties of the proposed system, and its accuracy and validation methods. *Conclusions:* The two most commonly used alternative modalities were IMUs and OPS. There was a lack of standardization among studies utilizing the same measurement modalities, as well as discrepancies in the methods used to assess performance. We provide a list of recommendations for future work to allow for more meaningful comparison between modalities as well as future research directions.

## 1. Introduction

Falls are a major public health issue and the leading cause of injury-related hospitalizations among seniors. According to the Canadian Institute for Health Information, in the 2017–2018 period, four out of five injury-related hospitalizations of individuals aged 65 and over were due to falls [1]. Twenty percent of falls can lead to head injuries or fractures triggering a sudden downward spiral in health [2], and falls are the most common cause for injury-related deaths in Canada among those aged 65 or above [3]. Falls may occur when an individual’s center of mass moves outside their base of support, and they are unable to react appropriately to recover from a balance loss. The loss of balance can be a result of a heel slip, toe slip, turning, or tripping [4]. Trips are the most common self-reported cause of outdoor falls [5] and it may happen if an individual fails to adjust their gait when negotiating obstacles or raised surfaces.

**Figure 1:**
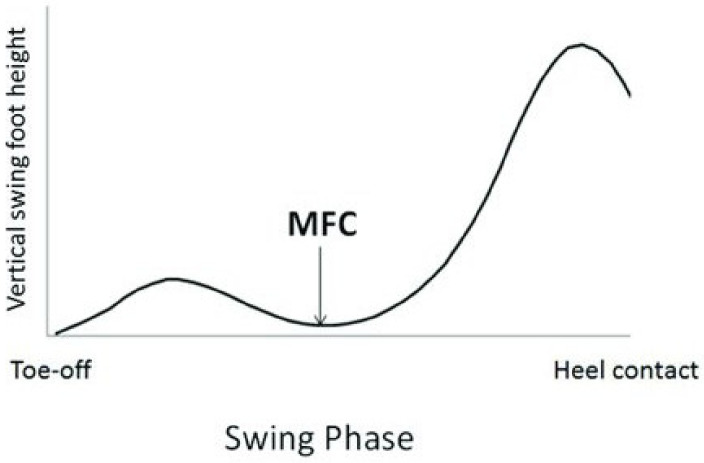
Vertical displacement of a point on the swing foot near the great toe over one stride. The minimum foot clearance (MFC) is defined by the local minima of the swing foot following toe-off. (Figure by Nagano et al. [9] licensed under CC by 4.0).

Figure 1 depicts the trajectory of a point on the foot over a gait cycle and the minimum foot clearance (MFC) point. The MFC point occurs at mid-swing phase where the distance between the lowest point on the swing foot and the ground reaches a local minimum and the base of support is small. Moreover, at this point, the foot has a forward velocity that is approximately three times faster than walking speed. Therefore, a trip occurring at, or near, the MFC point is more likely to lead to a loss of balance and/or fall than at other points in the gait cycle [6,7]. Individuals with smaller MFC values who do not lift their feet much when they walk are at higher risks of trip-related falls [8].

The risk of tripping is also dependent on the height of a given obstacle in the environment. Interestingly, different jurisdictions have different allowances for how large an obstacle can be. For instance, the Americans with Disabilities Act allows vertical changes in level (obstacles) on walkways up to 6.4 mm. If obstacles are between 6.4 and 13 mm, they are required to be beveled to reduce the risk of a trip [10]. In contrast, guidelines for the city of Toronto [11] allow level changes up to 13 mm with no intervention. One of our related projects is investigating our hypothesis that smaller level changes may even pose a greater threat than larger level changes for older adults with poorer vision since the smaller obstacles may be harder to see and avoid [12].

An accurate understanding of the MFC distribution for the general population would help estimate the risk posed by obstacles of different sizes in real-world settings. The data would make it possible to develop evidence-based guidelines for the design of the built environment to reduce the risk of falls and to protect the most vulnerable individuals. Optical motion capture systems that use a set of video cameras to track markers placed on participants are the gold standard for measuring gait parameters, including MFC [13]. They typically only track a small number of points on the feet, but virtual markers can also be added at additional points to measure MFC more accurately [14]. However, several limitations make it difficult to use optical motion capture systems for developing a real-world estimate of the general population’s MFC distribution. The processes for both data collection (camera calibration, marker placement) and data processing (labelling and filling in gaps in marker trajectories) with a motion capture system can be time-consuming and the amount of equipment involved makes it difficult to use these systems outside of a controlled laboratory environment. Collecting data outside the laboratory is desirables since controlled environments are known to influence the way participants walk compared to natural environments [15] and this can limit the number and types of participants that are included in studies.

We therefore performed a two-part scoping review to answer the following questions (RQs):

RQ1. What sensing modalities have been used for MFC measurement, other than optical motion capture systems?

RQ2. What are the reported level ground MFC values for ambulatory adults with functional limitations and what are the most common measurement modalities used in these assessments?

This paper focuses on answering RQ1 and reviews the existing research on alternative modalities to optical motion capture systems for measuring MFC, and error correction methods to increase the accuracy of existing alternatives. We also review the suitability of using these modalities and methods for the estimation of MFC values on outdoor walkways by comparing the performance, portability, and complexity of each system. A companion paper answers RQ2 and identifies conditions that impact MFC and the range of MFC values individuals with these conditions exhibit [16].

## 2. Methods

The review was done in accordance with the Preferred Reporting Items for Systematic Reviews and Meta-analysis extension for Scoping Reviews (PRISMA-ScR) reporting guidelines. A scoping review was chosen for this work based on the diversity of the technologies and experiment designs that we were reporting on, which would be inappropriate for a systematic review.

### 2.1. Information Sources and Search Strategy

To collect potentially relevant papers, the review was conducted in July 2019 in the following four databases: Medline, Embase, Compendex, and Web of Science collection. The search was not complemented with hand-searching or reviewing of reference lists.

The search strategy was reviewed by an experienced librarian/information specialist. The search term used in Medline was “((foot or toe? or heel?) adj2 (clear* or trajector*))” and was limited to human participants and the English language. The strategy was adapted accordingly to suit each database’s search requirements.

### 2.2. Eligibility Criteria

To be included in our review, the papers had to meet the following criteria: (1) be published in an academic journal or conference proceeding and (2) report on the assessment of MFC on level-ground surfaces, this criterion was chosen to ensure maximal uniformity that would allow for the potential comparison of MFC measurements between studies. It is important to note that we also included papers where the entire foot trajectory was measured as opposed to explicitly reporting only the MFC, since calculating MFC would be possible from foot trajectory data.

Papers were excluded if they were: intervention trials, studies with stairs, obstacle walking, or walking on sloped surfaces and studies that included children as participants. Furthermore, the papers were restricted to the English language and those that involved human participants. All papers published prior to July 2019 (when the search was conducted) were included.

Papers were placed into one of two categories corresponding to RQ1 and RQ2. Studies focusing on the measurement modality of MFC are discussed further in this paper. Studies focusing on MFC values and how it is affected by various conditions are discussed in our companion paper [16].

### 2.3. Selection of Papers

Upon completion of the initial search, abstract and full-text screening were conducted independently by two reviewers (GD and AA). In cases of conflict, a 3rd reviewer (TD) was recruited for resolution. The eligibility criteria proposed in Section 2.2 was used to guide all levels of screening. Data extraction was performed independently. The screening process was conducted using Covidence (Veritas Health Innovation, Melbourne, Australia), and the charting was done in Microsoft Excel (Microsoft Corporation, Redmond, Washington, DC, USA).

### 2.4. Data Charting and Analysis

A single reviewer performed data analysis following the extraction of the data. For each paper, the following paper characteristics were tabulated: title, whether the paper was published in a journal and the main technology used in their proposed system. Furthermore, the following parameters were tabulated following extractions: the sensors used in each paper, the employed validation system, the performance measures for the proposed system, the point of reference on the foot used to calculate clearance, the size of the proposed system, the characteristics of the proposed system (namely the method of data analysis, its ability to operate in real-time, and whether the system is a shoe-worn attachment).

### 2.5. Critical Appraisal of the Studies

The authors did not employ a ranked critical appraisal system to analyze the papers, given that the goal of the review was to simply capture all available technologies that measure MFC in the literature.

## 3. Results

### 3.1. Search and Selection of Articles

**Figure 2:**
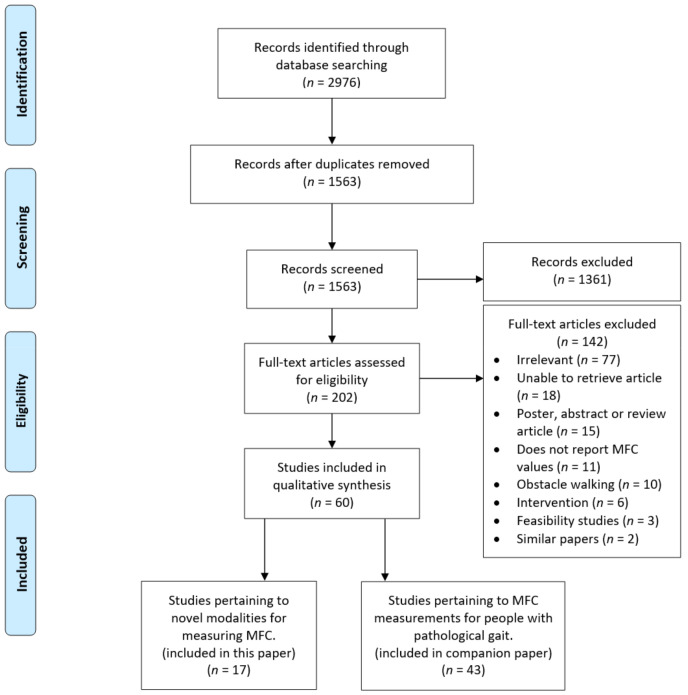
The flowchart of review process.

Figure 2 shows a summary of the search process including reasons for excluding studies at each stage. The initial search of the electronic databases identified 2976 potentially relevant titles. After the removal of duplicates, 1571 papers remained. After screening by title and abstract for inclusion and exclusion criteria, 202 papers were selected for full-text assessment. Of these, 17 unique papers, eleven journal articles, and six conference proceedings, were selected to be included in the category of papers answering our first research question. The articles were published between 1999 and 2019.

The sensor modalities explored in these studies were as follows, in order of frequency: inertial measurement units (IMUs) (58.8%), ultrasonic sensors (11.8%), infrared sensors (IR) (11.8%), optical proximity sensors (OPS) (5.8%), ultra-wideband sensors (UWB) (5.8%), and laser ranging sensors (5.6%). Table 1 lists a summary of the included papers’ characteristics.

### 3.2. Summary of the Papers

Table 2 summarizes the key finding of each paper. The extracted features are as follows: (1) ‘Sensors’ describes the sensors used in the developed system; (2) ‘Validated against’, describes the validation system used in each paper to compare the performance of the developed system; (3) ‘Clearance accuracy ± precision’ describes the performance of the developed system; (4) ‘Clearance point’ describes the point on the foot that is used to measure the distance between the foot and the ground; (5) ‘Dimensions’ describes the physical dimensions of the developed system; (6) ‘Real-time’ indicates if the system can calculate the parameters in real-time; (7) ‘Shoe-worn attachment’ indicates if the developed system can be considered a wearable attachment or not; and (8) ‘Data processing’ describes the methods used for processing the data gathered with the developed system.

We identified six different ways that foot clearance was measured. The most common method (*n* = 6) used a point on the mid-foot to measure the point of clearance. Four papers measured the clearance of the toe, four measured heel clearance, one measured minimum distance between the entire foot and the ground, and another one measured the clearance of the first metatarsal head. One paper measured toe and heel clearance simultaneously. Note that this information was either explicitly stated in the corresponding papers or was deduced based on the sensor placement on the foot. The papers in which the point of clearance was deduced are marked in Table 1 with †.

## 4. Discussion

### 4.1. Alternatives to Optical Motion Capture Systems

Our scoping review identified three families of sensor modalities that were used as alternatives to optical motion capture systems for the measurement of MFC. They were:

1. IMUs.
2. Using proximity sensors such as OPS, calculating time of flight using IR, UWB, ultrasonic, and laser ranging sensors.
3. Ultrasound sensors for 3D reconstruction of foot trajectory.

IMUs were the most common sensor modality used in the papers we found. In these papers, foot clearance values were estimated by double integrating acceleration data from these IMUs. All three modalities had the potential to be used as an alternative to optical motion capture systems for MFC measurement, since they all provide relatively good performance, portability, and complexity, as described in the following section.

### 4.2. Performance, Complexity, and Portability

**Figure 3:**
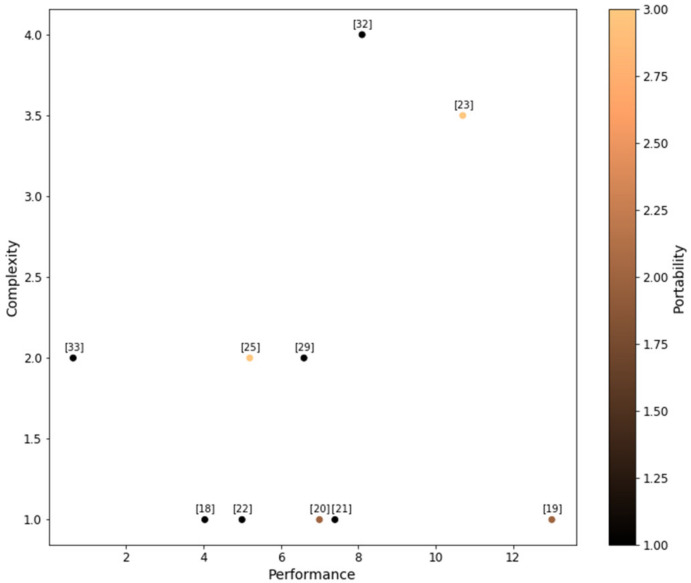
Comparing the identified systems in performance, complexity, and portability. Complexity score is determined based on the number of sensors used in the system. The error is calculated as RMSE or accuracy ± precision and the portability is determined by using a scoring system proposed by the authors. The numbers in square brackets refer to paper citations for the corresponding systems.

Among the 17 papers we identified, 10 reported the performance of their system in measuring foot clearance against gold standard (*n* = 9) or an ultrasonic measurement system (*n* = 1). Figure 3 compares these ten papers with regards to their performance, complexity, and portability.

To evaluate performance, we compared the reported error of these systems—either the use the Root Mean Square Error (RMSE) or accuracy ± standard deviation. To assess the relative complexity and portability, the two reviewers independently ranked the systems and resolved the conflicts with the third reviewer. The complexity was ranked on a scale of 1 to 5 (5 being the most complex system) based on the number of sensors the systems used. To evaluate portability, scores were assigned to papers based on answers to the following questions:

(1) Is the system mountable on the shoe? (2) Does the system have wireless communication? (3) Does the system include either an onboard or a portable processor?

Our approach was to score one point for each positive answer; zero points in case of negative answer, with a maximum of three points. Final portability scores range between 0 and 3 (3 being the most portable) and are represented by a color gradient in Figure 3. Almost all systems (16 out of 17 systems) were small in size and available as an attachment worn on the subject’s shoe/foot.

We also noted that a number of papers either did not compare their system against a gold standard at all, or compared it against systems that did not have well-defined performance themselves [24,26,27,28,31,33]. For instance, Yongbin Qi et al. [33], reported very low errors, but compared performance against an ultrasound system and failed to report the accuracy of their gold standard system. Another example is Wahab et al.’s study [31], which validated their system against a ruler.

Another difference that made it difficult to compare results of different studies was that not all studies used the same metrics to report the performance of their device. While many used accuracy± precision [19,22,23,29,32], some measured the performance of their device with RMSE [18,20], or correlation [30].

As seen in Figure 3, while the systems farther along the performance axis tend to score higher on the portability scale, we also notice that the more portable a system was, the worse it performed. The Wang et al. [26] paper, which was the only paper that tested their system outdoors, did not report any performance metrics on MFC measurement. Yongbin Qi et al. [33] reported the smallest error among these papers, showed great promise in the UWB technology. However, it is worth noting that Yongbin Qi et al. [33] validated their system against an ultrasound system, which is more prone to error compared to an optical motion capture system. Zhang et al. [25] have struck a good balance between portability, performance, and complexity. They used an instrumented footwear unit called SoleSound that can be inserted into the shoe as a sole and collected the data via a single IMU.

Several systems provided additional features that would be valuable for the development of a functioning, fully portable system for gait analysis. Wang et al. [26] used GPS data to cluster repeated paths participants walk in their daily lives. This allowed for a more focused and meaningful analysis of spatiotemporal gait parameters when the gait data is recorded in the real world. Using the IMU data, Ishikawa et al. [28] developed a method to classify the environment in which a participant walks. Benoussaad et al. [21] developed an algorithm wherein acceleration data only used to calculate distance measures and therefore it was robust to sensor misalignment. Tunca et al. [24] used “medial-lateral foot angular change detection” to detect gait events, which allows operation of the system under fewer assumptions of pathological gait.

### 4.3. Minimum Foot Clearance Definition

The definition of foot clearance was not consistent among the papers we reviewed. Different papers measured the distance to the ground from different points on the foot with most measuring the clearance of the mid-foot or the heel, none justified their choice and many omitted reporting where their measurement was taken. MFC, minimum toe clearance (MTC), and minimum heel clearance (MHC) represent three separate parameters that cannot be directly compared with each other. The MTC and MHC are considered the distance between the ground with the subject’s toe and heel, respectively, while MFC is the minimum distance between the ground and the subject’s foot, which may not occur at the toe/heel. We recommend the specific measurement used be stated explicitly and justified in future work. This improvement would allow for comparison between studies as well as for meta-analyses of foot clearance studies to provide guidance on design and maintenance standards for the built environment.

If the purpose of the gait study is to analyze or prevent trips, no single stationary point on the foot should be used to define tripping risk. According to Telonio et al. [34], all sections of the foot can potentially be the closest to the stairs during stair descent, meaning that MFC can happen in all the areas of the foot. Therefore, the MFC value, which is the distance between the lowest point of the foot and the ground is measured during swing phase, seems to be the best possible measure for this purpose.

### 4.4. Recommendations for Future Research

Our scoping review highlighted a number of gaps that results from a lack of standardization both in experimental methods and reporting standards. Here, we have provided recommendations for improving foot clearance studies along with some directions for future work:

1. Foot clearance studies should attempt to estimate the lower point on the foot by using multiple markers/virtual markers for more precise targeting of what is likely to be the lowest point on the foot. At a minimum, we recommend that authors in any particular study explicitly report the marker placement strategy to make their readers aware of possible bias. Future work may also benefit from using a number of markers on the foot to model a three-dimensional surface defining the bottom of the foot. The MFC value could then be estimated by measuring the minimum distance between this surface and the ground.
2. Many of the systems in this review were considered to be portable, but only one paper [26] carried out measurements in an outdoors setting. We recommend future work collect foot clearance data in outdoors settings, preferably with naïve participants with a wide range of ages and abilities that include the most vulnerable individuals in our population.
3. The majority of systems we identified utilized a single sensor modality on its own. Future work should consider using combinations of sensors to improve accuracy. For instance, proximity sensors could be used to correct drift errors that are common with IMUs.
4. Even though eleven studies measured their performance by comparing with optical motion capture systems, these gold standard systems may differ in accuracy due to differences in factors such as the number and resolution of cameras being used, distance of cameras to the participant, the size of markers used, and the amount of marker movement artifact present. Therefore, we recommend future work include validation against a gold standard system where the accuracy of the gold standard system (in mm) is reported to allow for more meaningful comparisons between studies.
5. Seven of the systems were capable of estimating the participant’s foot clearance in real-time. Future work should consider the potential of these systems being used to provide the wearer real-time feedback in the form of a prompt to increase foot clearance if the system detects an individual may be at risk of tripping.

### 4.5. Limitations

There are several limitations to our findings that future work should address. First, since our inclusion criteria limited our search to papers discussing the measurement of MFC over level ground in adults, we may have missed alternative modalities used in studies involving children, stair and obstacle walking, sloped surfaces, or intervention studies. Second, journals were not hand-searched through checking reference lists and unpublished or grey literature was also not included in our search. Finally, we did not employ a ranked critical appraisal system to analyze the papers we found.

## 5. Conclusions

Our scoping review identified 17 papers that discussed the use of modalities other than optical motion capture for measuring minimum foot clearance. The alternative modalities used were IMU sensors (*n* = 10), proximity sensors (*n* = 5), and ultrasonic sensors (*n* = 2). Our analysis showed that there was a lack of standardization among studies utilizing the same measurement modalities. We also found differences in the validation methods used and the performance metrics selected. Future study of foot clearance should further evaluate these alternative modalities, target more data collection in real-world settings and use standardized outcome measures to allow for more meaningful comparison between studies. Other recommendations include estimating the true lowest point of the foot by using multiple markers, carrying out more experiments in outdoor settings, using a combination of sensors to reduce error, reporting accuracy of the motion capture model that is being used for validation and further exploration the effect of providing real-time feedback to wearer in case of high tripping risk.

## Figures and Tables

**Table 1:** General characteristics of the included papers (Conf, conference proceeding; IMU, inertial measurement unit; IR, infrared sensor; OPS, optical proximity sensors; UWB, ultra-wideband sensors.).

Paper	Title	Journal/Conference	The Main Technology Discussed
Lai et al. [17]	Measuring toe clearance using a wireless inertial sensing device	ISSNIP (Conf)	IMU
Santhiranayagam et al. [18]	Estimation of endpoint foot clearance points from inertial sensor data	IEEE EMBS (Conf)	
Mariani et al. [19]	Heel and toe clearance estimation for gait analysis using wireless inertial sensors	IEEE Transactions on Biomedical Engineering	
Santhiranayagam et al. [20]	A machine learning approach to estimate Minimum Toe Clearance using Inertial Measurement Units	Journal of Biomechanics	
Benoussaad et al. [21]	Robust foot clearance estimation based on the integration of foot-mounted IMU acceleration data	Sensors (Switzerland)	
Kitagawa et al. [22]	Estimation of foot trajectory during human walking by a wearable inertial measurement unit mounted to the foot.	Gait & posture	
Minto et al. [23]	Validation of a footwear-based gait analysis system with action-related feedback	IEEE Transactions on Neural Systems and Rehabilitation Engineering	
Tunca et al. [24]	Inertial sensor-based robust gait analysis in non-hospital settings for neurological disorders	Sensors	
Zhang et al. [25]	Regression models for estimating kinematic gait parameters with instrumented footwear	IEEE Biorob (Conf)	
Wang et al. [26]	Analyzing gait in the real world using wearable movement sensors and frequently repeated movement paths	Sensors (Switzerland)	
Merat et al. [27]	A miniature multi-sensor shoe-mounted platform for accurate positioning	IEEE SMC (Conf)	Laser distance sensor + IMU
Ishikawa et al. [28]	Real-time foot clearance and environment estimation based on foot-mounted wearable sensors	IEEE IECON (Conf)	IR + IMU
Arami et al. [29]	An accurate wearable foot clearance estimation system: toward a real-time measurement system	IEEE Sensors Journal	
Kerr et al. [30]	Using an optical proximity sensor to measure foot clearance during gait: agreement with motion analysis	Journal of Medical Devices	OPS
Wahab et al. [31]	Development of shoe attachment unit for rehabilitation monitoring	Medical and Rehabilitation Robotics and Instrumentation	Ultrasonic
Qi et al. [32]	Ambulatory measurement of three-dimensional foot displacement during treadmill walking using wearable wireless ultrasonic sensor Nnetwork	IEEE Journal of Biomedical and Health Informatics	
Yongbin Qi et al. [33]	Using wearable UWB radios to measure foot clearance during walking	IEEE EMBC (Conf)	UWB

**Table 2:** The extracted information from the papers included in this review.

Paper	Sensor(s) Used	Validated Against	System Performance (Clearance Accuracy ± Precision or RMSE)	Clearance Point	Dimensions (L × W × H) (mm)	Real-Time	Shoe-Worn Attachment	Data Processing
Lai et al. [17]	Tri-axial accelerometer and tri-axial gyroscope	Motion capture system (Optotrak)	NR	Toe	NR	NR	Yes	Analysis: Double integration of acceleration Corrections: resetting position by using angular velocity to detect toe-off
Santhiranayagam et al. [18]	Tri-axial accelerometer and tri-axial gyroscope	Motion capture system (Optotrak)	RMSE: 4.04 mm	Toe	NR	Yes	Yes	Analysis: General Regression Neural Network trained with raw IMU sensor data
Mariani et al. [19]	Tri-axial accelerometer and tri-axial gyroscope	Motion capture (VICON, 7 cameras)	13 ± 9 mm	Toe	50 × 40 × 16	NR	Yes	Analysis: Kinematic model to simultaneously estimate toe and heel position. 3D trajectory reconstruction Corrections: linear de-drifting techniques
Santhiranayagam et al. [20]	Tri-axial accelerometer and tri-axial gyroscope	Motion capture system	RMSE: approx. 7 mm	Toe	NR	Yes	Yes	Analysis: General regression neural network trained from raw and integrated inertial signals
Benoussaad et al. [21]	Inertial measurement unit	Motion capture system (Vicon)	7.4 mm (normal walking)	Heel ^†^	NR	No	No	Analysis: Double integration, robust to sensor misalignment Corrections: 3D Frame Transformation and Gravity Removing, Foot-Flat Phase Detection, cancellation of in-stride drift
Kitagawa et al. [22]	Tri-axial accelerometer and tri-axial gyroscope	Motion capture (MAC3D, 8 cameras)	2 ± 7 mm	Mid-foot ^†^	37 × 46 × 12	No	Yes	Integrating the gravity-compensated translational acceleration during swing phase Corrections: Zero vertical velocity and displacement
Minto et al. [23]	2 IMUs (shank and foot) five vibrotactile transducers Four piezoresistive force sensors accelerometer pressure sensors	Motion capture system (Vicon, 10 cameras)	7 ± 3.7 mm	Mid-foot ^†^	NR	Yes	Yes	Analysis: Gait cycle detected by pressure sensors. Displacement calculated by double integration of acceleration data Corrections: Zero-velocity update
Tunca et al. [24]	Inertial sensor	Microsoft Kinect v2 and slow-motion camera	NR	Mid-foot ^†^	54 × 33 × 14	No	Yes	“Medial-Lateral foot angular change detection” which allows operation under less assumption of pathological gait
Zhang et al. [25]	Multi-cell piezo-resistive sensor and inertial measurement unit	Motion capture system (Vicon, 8 cameras)	Mean absolute error: SVR: 3.72 ± 0.87% LASSO: 3.00 ± 0.87%	Mid-foot ^†^	Shoe sole	YES (LASSO)	Yes	Analysis: Lasso regression and support vector regression
Wang et al. [26]	Inertial sensor, GPS receiver and barometric altitude sensor	NR	N/A	Mid-foot ^†^	70 × 48 × 26	No	Yes	Analysis: Isolating Repeated paths using GPS Corrections: Zero-Angular-Rate Update and Zero-velocity Update
Merat et al. [27]	microcontroller unit (MCU), an inertial measurement unit (IMU), three laser ranging sensors and a 4-in-1 barometer module	NR	N/A	Heel ^†^	32 × 20 × 10	No	Yes	Analysis: Tine of flight for distance estimation and IMU for orientation estimation Corrections: Error-state Kalman filter
Ishikawa et al. [28]	IMU + 2 IR sensors with different orientations	NR	N/A	Heel	NR	Yes	Yes	Analysis: IR for height of the sensor, IMU for orientation estimation Able to recognize the environment. Corrections: Kalman filter
Arami et al. [29]	Three configurations comprising one to three IR sensors and a configuration of single IR sensor and IMU	Motion capture system (Vicon, 11 Cameras)	Heel: 7.6 ± 0.0 mm (2/3 IR) Toe: 6.3 ± 3.3 mm (2/3/1+IMU IR)	Toe and heel	NR	Yes	Yes	Analysis: Calculating height of sensor by time of flight Orientation is estimated from difference in position and height of IR sensor or taken from IMU
Kerr et al. [30]	Optical proximity sensor Wireless transmitter	Motion capture system (Pro-reflex 500)	Not provided (high correlation)	First metatarsal head	NR	No	Yes	Distance from OPS sensor
Wahab et al. [31]	Ultrasonic sensor, Inertia Measurement Unit (IMU)	Ruler	NR	Heel ^†^	Modified-Phase-Only-Correlator	Yes	Yes	Analysis: Use ultrasound as a time-of-flight sensor IMU for orientation estimation
Qi et al. [32]	Ultrasound generator board	Motion capture system (8 cameras)	0.62 ± 7.48 mm	Heel ^†^	mobiles: transmitter and receiver: 40 × 16 controller board: 40 × 60	No	Partly	Analysis: Estimating location of mobiles based on distance from anchors. Using distance information to reconstruct 3D foot trajectory.
Yongbin Qi et al. [33]	Pair of wearable UWB transceivers	Ultra-sound system	0.64 mm	Heel ^†^	Width: 24	No	Yes	Analysis: Calculating height of sensor by time of flight Corrections: Modified phase-only correlator

^†^ The papers with the deduced point of clearance.
